# Society Meetings

**Published:** 1906-09

**Authors:** 


### SOCIETY MEETINGS.

The Association of Military Surgeons of the United States will hold its Fifteenth Annual Meeting in Buffalo, September 11, 12, 13, 14, 1906, under the following administration: president Lieutenant Colonel Albert Henry Briggs, surgeon 65th Regiment National Guard of New York; first vice-president, Brigadier General Robert M. O'Reilly, Surgeon General United States Army; second vice-president, Rear Admiral Presley M. Rixey, Surgeon General United States Navy; third vice-president, George Tully Vaughan, recently Assistant Surgeon General of the Public Health and Marine Hospital Service; secretary and editor, Major James Evelyn Pilcher, Captain (Retired) United States Army, Carlisle Pa.; treasurer, Major Herbert Alonzo Arnold, Surgeon in the National Guard of Pennsylvania, Ardmore, Pa.

The full staff of committees was published in the Journal for April, 1906.

---

DR. G. H. WESTINGHOUSE entertained the Medical Union of Buffalo at dinner at his summer home on Grand Island on Wednesday evening, July 25, 1906.

Supper was served on the lawn under widespreading trees. Among those present were Drs. S. S. Green, George L. Brown, A. H. Briggs, Vertner Kenerson, C. E. Congdon, W. Scott Renner, A. W. Bayliss, W. C. Krauss, Walter D. Greene, Ernest Wende, Edward J. Meyer, and Harry Mead. Dr. Winship, of Macon, Ga.; Dr. R. H. Satterlee and Dr. A. C. Howe were special guests.

---

THE EIGHTH DISTRICT BRANCH OF THE MEDICAL SOCIETY OF THE STATE OF NEW YORK, held its first meeting August 17 and 18, 1906, at the University Club, Buffalo, under the presidency of Dr. DeLancy Rochester, who was reelected for the ensuing year. Among the papers presented was one by Dr. Marshal Clinton, of Buffalo, entitled, The use of local anesthetics in operations on the chest and abdomen, which is to be published in a future issue of the Journal.

---

THE MISSISSIPPI VALLEY MEDICAL ASSOCIATION will hold its thirty-second annual meeting at Hot Springs, Ark., November 6-8, 1906, under the presidency of Dr. J. H. Carstens, of Detroit. Dr. Henry E. Tuley, of Louisville, the secretary, in his circular of announcement extends a cordial invitation to every physician in the Mississippi valley to attend the meeting. The headquarters of the association will be established at the Arlington Hotel.

---

THE BRITISH MEDICAL ASSOCIATION is holding its seventy-fourth annual meeting at Toronto, while these pages are going through the press, as announced in the August issue. A number of Buffalo physicians are in attendance.

---

THE INTERNATIONAL HOMEOPATHIC CONGRESS and the American Institute of Homeopathy will hold its sixty-second annual session at Atlantic City, N. J. September 10-16, 1906.

